# The placental RCAS1 expression during stillbirth

**DOI:** 10.1186/1477-7827-3-24

**Published:** 2005-06-17

**Authors:** Lukasz Wicherek, Marek Klimek, Artur Czekierdowski, Tadeusz J Popiela, Krystyna Galazka, Tomasz Tetlak, Andrzej Gilowski, Magdalena Dutsch-Wicherek

**Affiliations:** 1 Department of Gynecology and Infertility of Jagiellonian University, 23 Kopernik Str, 31–501 Krakow, Poland; 2 Ist Department of Gynecology of the Medical University in Lublin, 16 Staszica Str, 20–081 Lublin Poland; 3 Radiology Department of Jagiellonian University, 19 Kopernik Str, 31–501 Krakow, Poland; 4 Department of Pathomorphology Jagiellonian University, 17 Grzegórzecka, 31–531 Krakow, Poland; 5 ENT Department of Jagiellonian University, 2 Sniadeckich Str, 31–531 Krakow, Poland

## Abstract

**Background:** Independently of the fetal death cause the beginning and course of stillbirth is closely related with the growing cytotoxic activity at the maternal-fetal interface. RCAS1 participates in the inhibition of maternal immune response during pregnancy. The alterations of RCAS1 protein expression in placental cells seem to determine the beginning of the labor and participate in the placental abruption. The aim of the present study was to investigate RCAS1 expression in placentas obtained following stillbirths or normal term births. Methods: RCAS1 expression was evaluated by Western blot method with the use of monoclonal anti-RCAS1 antibody in 67 placental tissue samples. Pregnant women were divided into four groups according to the mode of labor onset – spontaneous or induced, and the type of labor, stillbirth or labor at term. Placental beta-Actin expression was chosen as a control protein. Relative amounts of placental RCAS1 were compared with the use of Student's t-test, whereas beta-Actin control data were compared with the use of Mann-Whitney U test. Results: The average relative amount of RCAS1 was significantly lower in women with induced stillbirths than in women with induced labor at term. Similarly, significantly lower RCAS1 placental levels were observed in patients with spontaneous stillbirths than in women with spontaneous labor at term. Significant differences in RCAS1 expression were also observed with the respect to the beginning of the stillbirth: spontaneous and induced. Lowest RCAS1 placental levels were observed in women with spontaneous stillbirth. Conclusions: These preliminary results indicate that the alterations of RCAS1 expression in the human placenta may be involved in the changes of maternal immune system that take place during stillbirth.

## Background

Dynamic fetal growth observed between 20th and 28th week of pregnancy is accompanied by fetal maturation which enables further uncomplicated extra-uterine growth. The fetal development is possible due to the phenomenon of maternal immunological tolerance to fetal antigens. Severe disturbances of the fetal growth might in some cases result in fetal intrauterine death. Some of the most common fetal death causes constituting about 50% of all cases include placental maturation disorders and fetal congenital malformations [1]. Stillbirth may be considered a form of complicated vaginal labor. Independently of the fetal death cause the beginning and course of stillbirth is closely related to the growing cytotoxic activity at the maternal-fetal interface. However, this activity does not have to arise simultaneously with the fetal death. In some situations fetal death may not be accompanied by immediate clinical features of the onset of labor. Molecular changes in membrane proteins expressed by trophoblast cells usually lead to the beginning of normal term delivery but may also be found in stillbirth. These proteins are neccessary for the development of maternal immune tolerance phenomenon. Cellular membrane proteins expressed by trophoblast cells that seem to be responsible for suppressing CTLs, dNK and NK cells are: Fas/Fas-L, killer inhibitory receptors family (KIRs), tumor necrosis factor receptors family (TNF) and others [2-4]. Recently, a novel factor called RCAS1 has been described [4-6]. RCAS1 is a type II membrane protein, expressed in extra-villous cytotrophoblast, villi-histiocytes, uterine endometrium and in various human cancer cells [7-12]. This protein acts as a ligand for a putative receptor that may be present on normal peripheral lymphocytes such as T, B, and NK cells. RCAS1 inhibits the growth of receptor expressing cells *in vitro *and *in vivo *and induces apoptotic cell death [13]. Main functions of RCAS1 include avoiding of immune recognition and evading immune surveillance by inhibition of maternal immune attack against fetal antigens [4,5]. The alterations of RCAS1 protein expression in placental cells seem to determine the onset of labor and participate in the placental abruption [5,6]. The aim of our study was to evaluate the changes of RCAS1 placental level during the stillbirths.

## Methods

### 2.1. The subjects

Relative amount of RCAS1 content was estimated in 67 placental tissue samples taken from normal term deliveries and from stillborn fetuses. Informed consent for the use of placental tissue was obtained from all patients. The approval from the Ethical Committee of the Jagiellonian University in Krakow (KBET/379/13/2003) for this research program was also granted. Stillborns were defined as dead-born fetuses either upon completion of 23 weeks of gestation, or when the fetal weight was over 400 grams. Autopsy was performed in Pathomorphology Department of Jagiellonian University in all cases. Based on the autopsy report review we included cases of intrauterine fetal death caused by fetal congenital malformations. According to the onset of stillbirth patients were devided into four groups. The first group included 6 women in whom labor was induced following the recognition of the intrauterine fetal death. The second group consisted of 5 women with stillbirths with spontaneous onset of labor. Two control groups consisted of pregnant women with normal term birth (37–43 completed week' gestation), and were selected according to the onset of labor, that was spontaneous (group III) or induced (group IV). The former group consisted of 31 pregnant women with spontaneous onset of vaginal deliveries preceded by the regular uterine contractions during first and second stage of the labor. The latter group included 25 women with induced vaginal term deliveries. Maternal characteristics of the women are shown in Table 1. Patients with preterm deliveries, chorioamnionitis, hypertension, diabetes mellitus and multiple pregnancies were excluded. All placentas were histologically examined by an experienced pathologist.

**Table 1:** The characteristics of subjects

Pregnant women (n = 67)	Maternal age ± SD (y)	Gestational age ± SD (wk)	Nulliparous (%)	Birth weight ± SD (g)	Mean Apgar ± SD
Induced stillbirths (n = 6)	28.5 (± 5.24)	25.23 (± 1.36)	33.3	612.6 (± 172.2)	-
Spontaneous stillbirths (n = 5)	27 (± 5.91)	25.57 (± 1.43)	40	650 (± 145.4)	-
Induced labor at term (n = 25)	28.07 (± 4.55)	38.96 (± 1.83)	60	3290 (± 467)	9.34 (± 2.02)
Spontaneous labor at term (n = 31)	28.16 (± 6.04)	39.68 (± 2.36)	58	3138 (± 649)	9.5 (± 1.22)

SD – standard deviation; (n – number of tissue samples)

### 2.2 Preparation of tissue extracts

Tissue samples (app. 0.5 × 0.5 × 0.5 cm) were obtained from the central part of the placenta collected following delivery and stillbirth and were immediately frozen. The 0.5 cm thick tissue samples contained villous lamina (extra-villous cytotrophoblast cells and syncytiotrophoblast) and cells from maternal decidua compacta (the external part of decidua basalis). The specimens were mixed with complete proteinase inhibitor cocktail (Roche, Germany) and homogenized on ice-bath in glass-glass Potter-Elvejhem homogenizer. The resulting suspensions were mixed with equal volume of SDS sample lysis buffer (4% SDS, 20% glycerol, 125 mM Tris-HCl pH 6.8) and boiled on water bath by 5 minutes. The chilled samples were then centrifuged at 16,000 g for 15 min at room temperature. The collected supernatants were used for further analysis.

### 2.3. Western blotting

Total protein content in the obtained supernatants was measured using BCA assay kit and different sample volumes (usually in the range of 2–10 μl) equivalent to 50 μg of total protein were then loaded on SDS-PAGE tris-tricine peptide-separating gels. Prestained broad range molecular weight proteins standard (Bio Rad, USA) was used in gel marker lane. Following electrophoresis the gels were electrotransferred on Immobilon-P polyvinylidene difluoride (PVDF) 0.45 μm membrane (Millipore, USA) in the buffer containing 10 mM 3-[cyclohexyloamino]-1-propanesulfonic acid (CAPS) pH 11, supplemented with 10% methanol. The obtained membrane blots were blocked overnight by gentle agitation in 5% bovine albumin in TST buffer (0.01 M Tris-HCl, pH 7.4, 0.9%NaCl), 0.5% Tween-20). All described procedures were performed at room temperature. Albumin solution was discarded and the membranes were then agitated for 2 hrs in TST with the mouse monoclonal anti-RCAS1 IgM-class antibody, 1: 4 000 dilution (Medical and Biological Laboratories, Japan). The membranes were then subjected to 4 cycles of washings in TST, 5 minutes each and immersed for agitation in the 1 : 2 000 dilution of SIGMA biotinylated anti-mouse IgM μ-chain specific antibodies for a 2 hours period. After 4 cycles of washings the membranes were then treated for 2 hours in 1 : 2 000 dilution of ExtrAvidin alkaline phosphatase conjugate (SIGMA, USA) and finally washed 2 times in TST and 2 times in TST without Tween-20. Color reaction was developed with the use of Fast Red TR/Naphtol AS-MX tablet set (SIGMA, USA). A sufficient bands intensity was obtained usually following 5 min period of developing. Obtained immunoblotts were then rinsed with distilled water and air-dried. Detailed description of the tissue preparation and semi-quantitative assessment of RCAS1 and control beta-Actin relative amounts in the tissue samples using Western blot technique has been presented in our previous reports [5,6]. The RCAS1 antigen was identified as a 32 kDa band and β-actin represented a 42 kDa band [6,10,11].

### 2.4. Computer-aided image analysis

Fluor-S MultiImager (BioRad, USA) was used to scan immunoblotted membranes and a QuantityOne software (BioRad, USA) was used to quantitate band intensities. All calculations were performed on RCAS1 antigen band having molecular mass of about 32 kDa [6,10,11]. The intensities of this band were expressed in arbitrary relative units and one unit (U) was defined as band intensity produced in the reference sample. This reference sample was randomly chosen but was strictly the same on all blots and was applied always in the same amount. Typical procedure of RCAS1 quantitation was as follows: scanned immunoblot membrane contained one lane of molecular mass standards, while one lane of sample used as a reference to calibrate RCAS1 amount and 12 lanes containing unknown samples. The location of 32 kDa RCAS1 bands in reference and unknown lanes was identified according to the lane containing molecular mass standard. The 32 kDa RCAS1 band intensities in reference and in unknown lanes were then calculated and expressed in pixel number units. These units were divided by pixel number of reference lane band which resulted in relative intensity units "U". The resulting intensity of reference lane band was always 1.00 U while the intensities of bands from 12 unknown sample lanes on the same membrane changed according to RCAS1 level (e.g., if RCAS1 amount in a given sample was 2 times higher than this in reference sample the result was 2.00 U. If the RCAS1 amount was 2 times lower then the result was 0.50 U). As mentioned earlier, because all immunoblots contained the same RCAS1 quantity standard and all lanes were loaded with the same amount of total protein (50 μg), the determined values were highly repetitive and independent of experiment conditions. The RCAS1 total amount in examined tissue samples was recognized relatively, which was necessary because no RCAS1 standard is available. The result always shows relative amount of RCAS1 32 kD antigen in 50 μg of total sample protein [5,6].

### 2.4. Statistical analysis

The distribution of the subjects was analyzed with the use of Shapiro-Wilk's test. RCAS1 were compared with the use of Student's t-test whereas β-Actin control data were compared with the use of Mann-Whitney U test. Differences between studied groups were considered significant at p < 0.05.

## Results

The presence of RCAS1 expression was evaluated in all placental tissue samples derived from vaginal deliveries at term and stillbirths. The relative amount of β-Actin in all groups were found to be identical (table 2), this indicate that equal loading of proteins was performed and allow a comparative study between RCAS1 expression in each group.

**Table 2:** The comparison of RCAS1 average relative placental amount assessed by Western blot method during vaginal delivery according to the course of the labor.

	RCAS1 placental relative amount (mean ± SD)	β-Actin placental relative amount (mean ± SD)
Spontaneous stillbirths (n = 5)	**0.2102 **(± 0.0601)	**1.1383 **(± 0.1438)
Induced stillbirths (n = 6)	**0.3334 **(± 0.1387)	**1.0924 **(± 0.1316)
Induced labor at term (n = 25)	**0.7131 **(± 0.2896)	**1.1559 **(± 0.7445)
Spontaneous labor at term (n = 31)	**0.4775 **(± 0.2728)	**1.1206 **(± 0.5316)

SD-standard deviation; (n – number of tissue samples)

The average relative amount of RCAS1 was significantly lower in placental tissue samples obtained from induced stillbirths than from samples collected following induced labor at term (p = 0.004). Also, average RCAS1 expression observed in placental tissue samples from women with spontaneous stillbirths was significantly lower than placental protein expression in samples from women with term labor with spontaneous beginning (p = 0.03). Significantly higher RCAS1 placental levels were found in women with induced labor when compared to women with spontaneous onset of labor at term (p < 0.001). Significant differences in RCAS1 expression were also observed within the group of stillbirths with the respect to the beginning of the stillbirth: spontaneous and induced (p = 0.06).

## Discussion

In order to investigate the possible role of RCAS1 in initiation of stillbirth, the expression levels of this protein were determined and compared between groups of patients with spontaneous and induced onset of stillbirth. The RCAS1 expression observed during spontaneous stillbirth was lower than the level of this protein found in induced stillbirth. Immune activity alterations noted during gestation have a local range and concern the endometrium-associated lymphoid tissue. Trophoblast cells participate in this phenomenon. Syncytiotrophoblast is terminally differentiated to functional trophoblast. The ratio of syncytiotrophoblast to cytotrophoblast increases with advancing gestation and at term syncytiotrophoblast occupies almost the entire placenta. The differentiation from cytotrophoblast to syncytiotrophoblast is identical to placenta development [14]. The cells of syncytiotrophoblast and cytotrophoblast play an important role in the function of the feto-maternal unit. The decrease of immune tolerance at term leads to the beginning of spontaneous labor, earlier appearance of this phenomenon may lead to miscarriage or preterm labor [15].

The activation of endometrium-associated lymphoid tissue is provided mainly by lymphocyte-mediated cytotoxicity. Normal endometrium is infiltrated predominantly by T lymphocytes, NK cells and macrophages. Increased number of B lymphocytes which constitute a small percentage during physiological cycle changes in endometrium is observed only in endometritis [16]. The analysis of mononuclear cells distribution in deciduas of term placentae revealed significantly increased number of dNK, NK and macrophages [17].

Our previous studies confirmed the observation of decreased RCAS1 expression level during labor of spontaneous onset when compared to induced labor at term. The decreased RCAS1 expression level seems to confirm the observation of higher dNK, NK activity, which might be clinically observed in the beginning of spontaneous labor, preterm labor or stillbirths [5]. Clinically asymptomatic onset of stillbirth indicates that that fetal demise might not be immediately recognized by maternal immune system. Lower placental RCAS1 expression in women with spontaneous beginning of stillbirth in comparison to induced stillbirths observed in our study seems to confirm this hypothesis. A correlation between RCAS1 expression and the infiltration by dNK cells in the human uterus found in early stage of pregnancy was observed by Oshima et al. [4]. In this study, in cases without maternal rejection RCAS1 was expressed mainly in trophoblast. In contrast, in cases with maternal rejection the expression of RCAS1 decreased strikingly. These changes were also accompanied by marked infiltration and activation of dNK cells. Normal placentas at term were selected as the controls. Immunohistochemical localization revealed the presence of RCAS1 in normal placental tissues at term. Positive staining for RCAS1 was found predominantly in extravillous cytotrophoblast and syncytiotrofoblast [4]. Therefore, it is possible that the onset of stillbirth may also require the decrease of immune tolerance which may reflect an increasing cytotoxic activity.

## Conclusion

Our results indicate that alterations in RCAS1 expression may be involved in the changes of maternal immune system that take place during stillbirth.

## List of abbreviations

RCAS1 (receptor-binding cancer antigen expressed on SiSo cells); CTLs (cytotoxic T lymphocytes); dNK (decidual natural killer); KIRs (killer inhibitory receptors); TNF (tumor necrosis factor).
